# Genome-Wide Dissection of the *CRF* Gene Family in *Brassica napus* Indicates that *BnaCRF8s* Specifically Regulate Root Architecture and Phosphate Homeostasis against Phosphate Fluctuation in Plants

**DOI:** 10.3390/ijms21103660

**Published:** 2020-05-22

**Authors:** Sheliang Wang, Hao Zhang, Lei Shi, Fangsen Xu, Guangda Ding

**Affiliations:** Microelement Research Centre, National Key Laboratory of Crop Genetic Improvement, Key Laboratory of Arable Land Conservation (Middle and Lower Reaches of Yangtze River), Ministry of Agriculture and Rural affairs, Huazhong Agricultural University, Wuhan 430070, China; sheliangwang2017@mail.hzau.edu.cn (S.W.); hao.zhang@webmail.hzau.edu.cn (H.Z.); leish@mail.hzau.edu.cn (L.S.); fangsenxu@mail.hzau.edu.cn (F.X.)

**Keywords:** *Brassica napus*, cytokinin response factor, expression profile, *CRF8*, root architecture, Pi homeostasis, *Arabidopsis*, *Nicotiana*

## Abstract

Phosphorus (P) is an essential macronutrient required for plant growth and development. The involvement of cytokinin response factors (CRFs) in phosphate (Pi) homeostasis and lateral root (LR) initiation in *Arabidopsis* has been revealed. However, little is known in oil crops. Here, we performed genome-wide dissection of the *CRF* family in *Brassica napus* to identify 44 members, which were evolutionally classified into 6 subgroups. Among them, four *BnaCRF8* genes were strongly upregulated by P deprivation, and were selected to be further investigated. Time course qRT-PCR analyses showed that four *BnaCRF8* genes were enhanced dramatically after 12 h of P stress. Analyses of the subcellular localization in tobacco leaves indicated that BnaA7.CRF8 and BnaC2.CRF8 were localized in the nucleus. The expression of *BnaCRF8* genes had constant negative effects on primary root growth and LR initiation and growth, and it reduced Pi acquisition and plant growth in *Arabidopsis*. Moreover, the expression of Pi homeostasis-related genes was modulated in *BnaA7.CRF8* overexpression plants. These results suggest that *BnaCRF8* genes might negatively regulate root architecture and plant growth through transcriptional modification of Pi homeostasis-related components. Overall, this study suggests that upregulation of *BnaCRF8* genes might be a smart adaptive strategy to cope with continuous Pi deficiency in the environment.

## 1. Introduction

Phosphorus (P) is an essential macronutrient required for plant growth and development. P-containing molecules, such as nucleic acids, phospholipids, and ATP, participate in many physiological and metabolic processes, including signal transduction and the regulation of protein phosphorylation [1]. The bioavailability of utilizable inorganic phosphate (Pi) in soil usually cannot satisfy the demand of plants for their optimal growth [2]. Thus, a series of Pi starvation responses (PSRs), such as changes in the root morphology and architecture, induction or enhancement in the expression of high-affinity Pi transporters, changes in lipid modification, and exudation of organic acids and phosphatases into the soil, have evolved to adapt to Pi stress [3,4]. Pi limitation has been shown to upregulate the expression of two *PHOSPHATE TRANSPORTER1* (*PHT1*) genes *PHT1;1* and *PHT1;4*, and cause the accumulation of their proteins on the plasma membrane to facilitate Pi uptake in plants [5]. *MiR399s* are upregulated by Pi stress to reduce *PHOSPHATE2* (*PHO2*) gene expression, resulting in the stability of PHOSPHATE1 (PHO1), which is responsible for Pi loading into the xylem in roots and translocation to the shoots [6,7,8]. *PHO2*, encoding the ubiquitin-conjugating E2 enzyme 24; interacts with PHT1;1, PHT1;4, and PHO1 proteins; and mediates their ubiquitination for degradation in cells [8]. Under Pi stress conditions, in order to reduce the demand for P in lipid metabolism, the concentration of phospholipids decreases and that of P-free lipid components, such as galactolipid, sulfoquinovosyldiacylglycerol (SQDG), and digalactosyldiacylglycerol (DGDG), increases [3]. Therefore, the lipid remodeling-related genes, including *SQD1* and *SQD2* (encoding enzymes in sulfolipid biosynthesis), *PLDζ1* and *PLDζ2* (encoding enzymes in phosphatidylcholine hydrolysis), and *DGD1* and *DGD2* (encoding digalactosyldiacylglycerol synthases), are induced by Pi depletion [9,10,11]. Furthermore, the *LOW PHOSPHATE ROOT1*/*2* (*LPR1*/*2*) genes, which encode multicopper oxidases, have been identified as important regulators of meristem responses to low Pi availability in the primary root (PR) of *Arabidopsis* [12], while the *PHOSPHATE ROOT DEVELOPMENT* (*PRD*) gene is involved in the regulation of root architectural responses to Pi starvation by controlling PR and lateral root (LR) elongation [13]. Moreover, many Pi starvation responsive transcription factors (TFs), such as *PHOSPHATE STARVATION RESPONSE1* (*PHR1*) and *PHR1-Like1* (*PHL1*), have been identified and shown to positively or negatively regulate several subsets involved in Pi homeostasis, as well as in root architecture modulation [3,14,15].

The plant hormone cytokinin regulates numerous growth and developmental processes. The cytokinin response factors (CRFs) have been identified as cytokinin-regulated TFs [16]. CRFs belong to the B-5 subgroup of the ethylene response factor (ERF) subfamily of the APETALA2/ETHYLENE RESPONSIVE FACTOR (AP2/ERF) TF superfamily [17]. AP2/ERF proteins comprise one of the largest TF families in plants and are defined by the presence of an AP2 DNA binding domain of around 68 amino acids [18]. A total of 12 *CRF* genes in *Arabidopsis* have been identified depending on a clade-specific approximately 65-amino acid CRF domain in the N-terminal region [16,18]. Among them, the *CRF8* gene is induced specifically in Pi-deprived *Arabidopsis* roots and shoots, and a knockout mutant of the *CRF8* gene phenotypically augments LR development, with higher Pi accumulation in plants [19]. In contrast, overexpression (OE) of *AtCRF8* in *Arabidopsis* reduces both the PR length and LR initiation [19]. Besides *AtCRF8*, another two *CRF* genes (*AtCRF2* and *AtCRF3*) are also involved in LR initiation and formation [20], while *AtCRF1*, *AtCRF2*, and *AtCRF5* affect the development of the cotyledon, leaf, and embryo [16].

Allotetraploid rapeseed (*Brassica napus*, AACC, 2n = 38), which is formed by natural hybridization between *B. rapa* (AA, 2n = 20) and *B. oleracea* (CC, 2n = 18) [21], is widely cultivated and has become the world’s second leading crop source of vegetable oil (following soybean) for human consumption. Although the *CRF* genes in *Brassica* crops, including *B. napus*, have been identified and their evolutionary relationship has been discussed [22], the Pi homeostasis-related *CRF* genes in *B. napus* have not been assessed. Furthermore, their molecular function in plant Pi homeostasis remains unknown. In this study, we identified 44 *CRF* genes, including 4 novel genes in the *B. napus* genome, and estimated their transcriptional responses in Pi stress. The four low Pi-responsive *BnaCRF8* genes were further investigated for their involvement in Pi homeostasis using OE transgenic *Arabidopsis* lines. Our work provides a comprehensive insight into the effects of *BnaCRF* genes on Pi homeostasis.

## 2. Results

### 2.1. Identification and Phylogenetic Relationships of the CRF Family in Brassica napus

**Figure 1:**
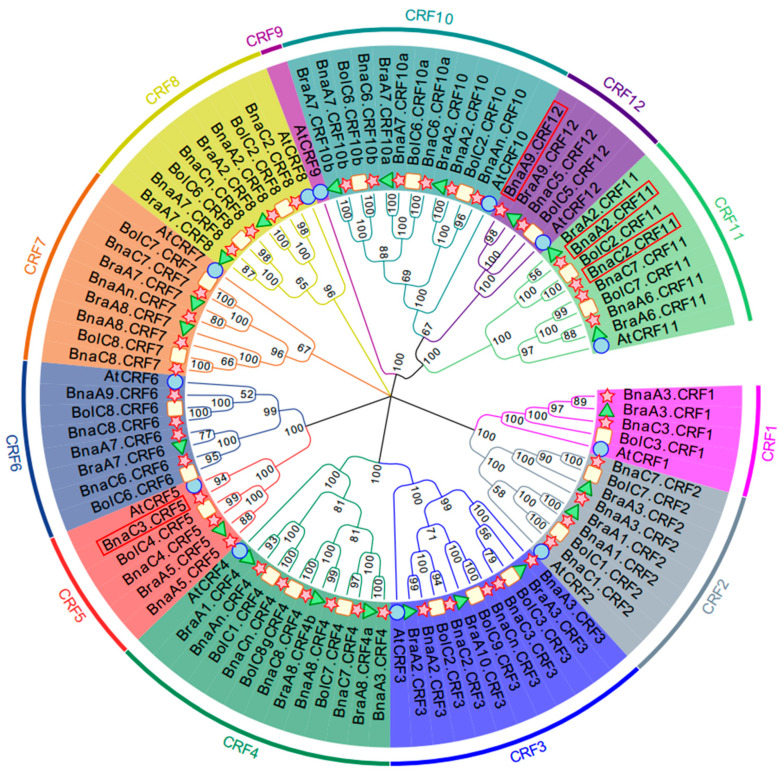
Phylogenetic tree of cytokinin response factor (CRF) genes in *Arabidopsis thaliana* and *Brassica* crops. Homologous genes of each *CRF* are distinguished by different colors. The unrooted tree was generated using ClustalW in MEGA7.0 using the full-length amino acid sequences of the 9, 44, 21, and 22 CRF proteins in *A. thaliana* (circle), *B. napus* (star), *B. rapa* (triangle), and *B. oleracea* (rectangle), respectively. Four *BnaCRF* genes newly identified in this research are highlighted with a red box.

To obtain the *CRF* family TFs in *B. napus*, we used the CRF protein sequences identified in the *Arabidopsis* genome to perform a BLASTP search against the *B. napus* genome. *Arabidopsis* has 12 *CRF* genes, and accordingly a total of 44 *CRF* genes were identified in the whole genome of *B. napus*. Except *AtCRF9*, the orthologous number of each *AtCRF* varied from 2 to 6 in *B. napus* (Table S1). To investigate the phylogenetic relationship of these *BnaCRF* genes, 21 and 22 *CRF* genes were identified from *B. rapa* and *B. oleracea*, respectively. Based on these CRF amino acid sequences, we generated a phylogenetic tree using the neighbor-joining (NJ) method (Figure 1). All *CRF* genes were evolutionally classified into six subgroups in which *CRF1* and *CRF2* were in subgroup 1, *CRF3* and *CRF4* were in subgroup 2, *CRF5* and *CRF6* were in subgroup 3, *CRF7* was in subgroup 4, *CRF8* was in subgroup 5, and *CRF9*, *CRF10*, *CRF11*, and *CRF12* were in subgroup 6. Most of the *BnaCRFs* located in the A and C subgenomes were consistently inherited from *B. rapa* and *B. oleracea*, respectively. However, the homologues of *BraA10.CRF3* disappeared in *B. napus*, and *BnaC3.CRF5* and *BnaA9.CRF6* were additionally duplicated after hybridization between *B. rapa* and *B. oleracea*. The genes of *CRF1*, *CRF3*, *CRF4*, *CRF7*, *CRF8*, *CRF10*, and *CRF12* suffered duplication after speciation of *Arabidopsis* and *Brassica* crops. The gene duplication events of *CRF2*, *CRF5*, *CRF6*, and *CRF11* in *Brassica* crops were either after or parallel to *Arabidopsis* formation. *CRF10* and *CRF12* resulted from the duplication of *CRF11*, then they in turn expanded independently in *Brassica* crops.

### 2.2. Gene Structure, Protein Features, and Chromosomal Duplication of the CRF Family in Brassica napus

**Figure 2:**
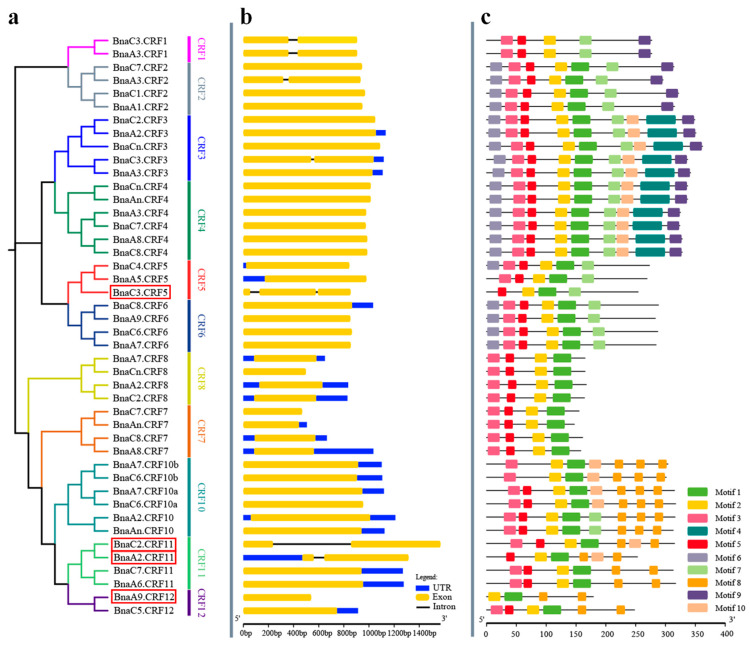
Phylogenetic relationships, gene structure, and architecture of conserved protein motifs in *CRF* genes from *Brassica napus*. (**a**) Phylogenetic relationships of *BnaCRF* genes. The phylogenetic tree (left panel) was constructed with MEGA 7.0 using the neighbor-joining (NJ) method with 1000 bootstrap replicates. Different homologous gene clusters are shown in different colors; (**b**) Gene structure of *BnaCRF* genes. Blue boxes indicate untranslated 5′- and 3′-regions; yellow boxes indicate exons; black lines indicate introns. Scale bar represents gene length; (**c**) Motif characterization of BnaCRF proteins. The motifs, number 1–10, are displayed in different colored boxes. More sequence information for each motif is provided in Figure S1. The scale bar represents the protein length. Four *BnaCRF* genes newly identified in this research are highlighted with a red box. Motif 3 indicates the CRF domain, while motif 2 together with motif 1 indicate the APETALA2/ETHYLENE RESPONSIVE FACTOR (AP2/ERF) domain.

To analyze the gene structure of *BnaCRFs*, the genomic sequences identified above were schematically organized (Figure 2). The full length of the exons of all members ranged from 444 to 1569 bp (Table S1). Six *BnaCRFs* were identified with a single intron, in which the intron length of *BnaC2.CRF11* was significantly longer than the others (Figure 2 and Table S1). *BnaC3.CRF5* exclusively had two introns, although they were small. The remaining 37 *BnaCRFs* were identified consistently with one exon (Figure 2 and Table S1). The putative BnaCRF proteins varied in length from 147 to 351 amino acids (Figure 2 and Table S1). The deduced BnaCRF amino acid sequences were further analyzed for comprehensive prediction of the conserved motifs using the multiple expectation maximization for motif elicitation (MEME) suite [23]. A total of 10 conserved motifs were queried in BnaCRF proteins (Figure 2). Of these, motifs 1 and 2 found in all submitted members were AP2/ERF domains, while motif 3, representing the CRF domain, was present in 41 BnaCRF proteins but not in BnaA5.CRF5, BnaA2.CRF11, and BnaA9.CRF12 proteins (Figure 2 and Figure S1). In addition, we observed that evolutionally close CRFs displayed a similar composition of motifs although more or less different (Figure 1 and Figure 2). The divergence in the composition of motifs and sequence length among BnaCRF proteins might determine their functional differentiation. It is important to note that both the AP2/ERF domain and CRF domain were conserved between *B. napus* and *Arabidopsis* (Figure S1). Further analyses of the physicochemical properties showed that the molecular weight (MW) of BnaCRF proteins ranged from 16.26 to 41.18 kDa, while the isoelectric point (PI) varied from 4.5 to 10 (Table S1). Chromosomal location analyses revealed that half of the *CRF* genes were distributed on the chromosomes of the A subgenome except A04 and A10. Similarly, the remaining 22 members were distributed on the chromosomes of the C subgenome except C04 and C09 (Table S1 and Figure S2). Furthermore, we identified 24 segmental duplication events but no tandem duplication events within the *BnaCRF* gene family by the BLASTP and MCScanX methods (Figure S3), indicating that segmental duplication events played a major driving force for the expansion of the *CRF* family in *B. napus*.

### 2.3. Expression Profiles of the BnaCRF Genes in Response to Phosphorus Stress

**Figure 3:**
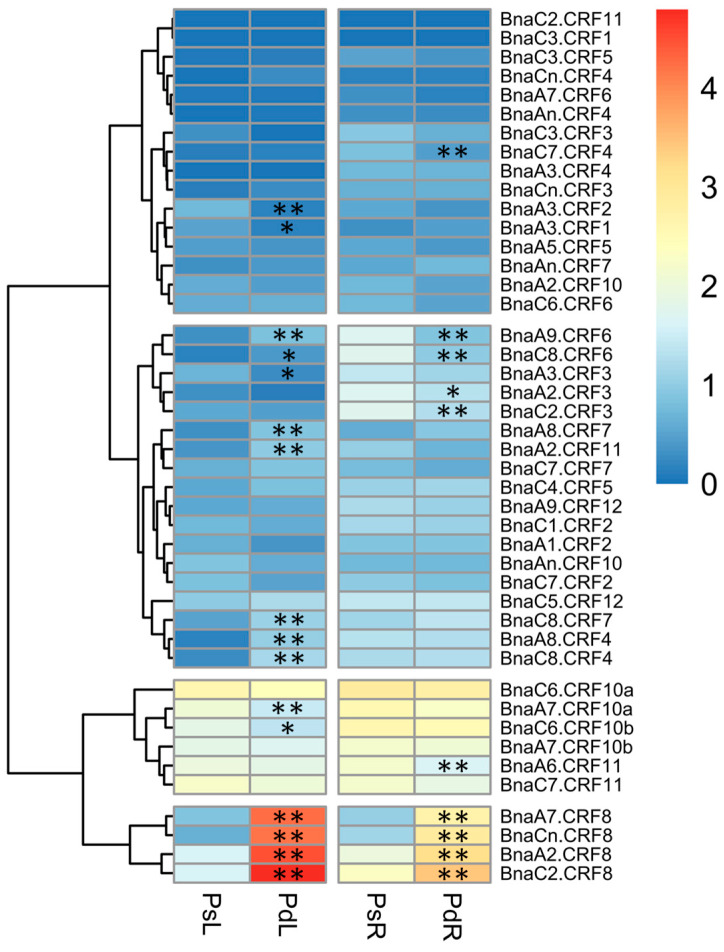
Expression profiles of the *CRF* gene family in response to phosphorus (P) stress in *Brassica napus*. Shoots and roots of P stress-treated plants were sampled for RNA-seq. The fragments per kilobase of exon per million mapped reads (FPKM) values of the 44 *BnaCRF*s were extracted and were converted into the logarithmic values. PsL: P-sufficient leaves; PdL: P-deficient leaves; PsR: P-sufficient roots; PdR: P-deficient roots. Asterisks indicate significant differences at * *p* < 0.05 and ** *p* < 0.01.

**Figure 4:**
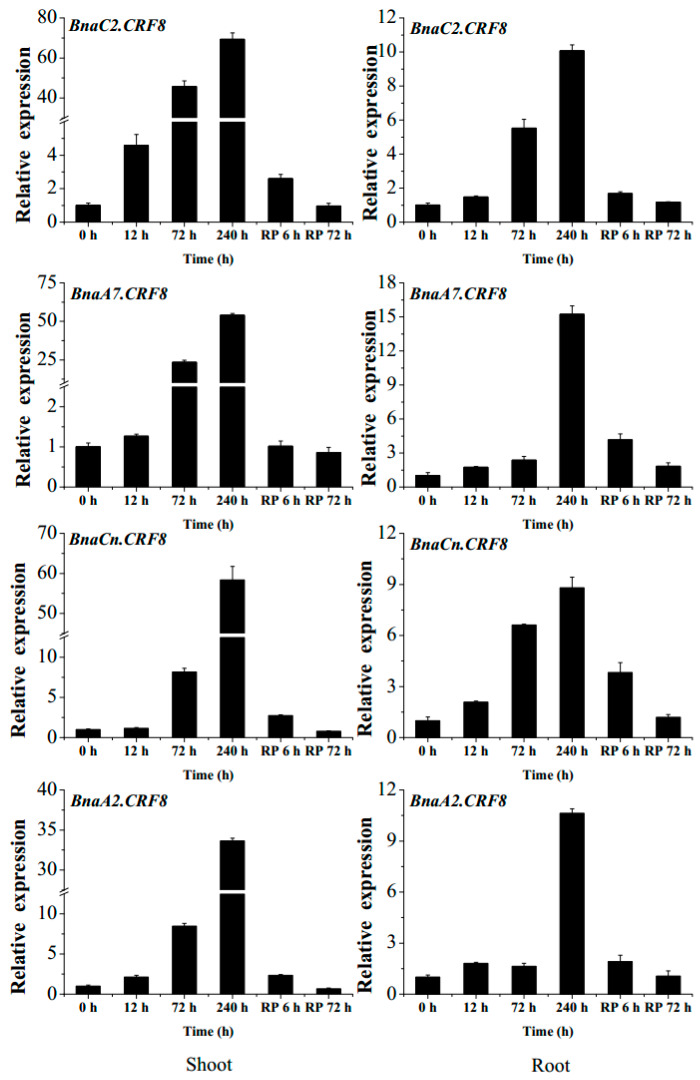
The expression changes of the 4 *BnaCRF8* genes in response to phosphate (Pi) deficiency and recovery in *Brassica napus*. Fifteen-day-old plants were treated with Pi starvation (0 μM Pi) for 10 days before Pi recovery (RP). Then, the shoots and roots of the plants were sampled separately, and qRT-PCR was performed at 6 time points (0, 12, 72, and 240 h after Pi starvation, and 6 and 72 h after RP). Values represent mean ± standard deviation (SD) of 3 biological replicates with 4 plants each.

Given the important roles of *CRFs* in nutrient stress regulation [19,24], we tried to identify the critical *BnaCRF(s)* in response to P stress. The expression profiles of the 44 *BnaCRF* genes were investigated by the RNA-seq assay of the *B. napus* seedlings treated with or without P. The fragments per kilobase of exon per million mapped reads (FPKM) values of the *BnaCRF* genes were extracted from the RNA-seq data and were converted into logarithmic values shown by a heat map (Figure 3). In the roots, *BnaC7.CRF4*, *BnaA9.CRF6*, *BnaC8.CRF6*, *BnaA2.CRF3*, *BnaC2.CRF3*, and *BnaA6.CRF11* were downregulated by P stress, while four *BnaCRF8* (*BnaA2.CRF8*, *BnaC2.CRF8*, *BnaA7.CRF8*, and *BnaCn.CRF8*) genes were strongly upregulated by P stress. In the leaves, the expression levels of five genes, including *BnaA3.CRF2*, *BnaA3.CRF1*, *BnaA3.CRF3*, *BnaA7.CRF10a*, and *BnaC6.CRF10b*, declined significantly under the low-P condition compared to that under the P-sufficient condition, while 11 genes were largely upregulated by P limitation, including the 4 *BnaCRF8* genes, which have the highest expression levels under P deprivation among all the *CRF* family members. To validate the expression profile of the four *BnaCRF8* genes, we performed the qRT-PCR assay with treatments of P deficiency for different times followed by P recovery (Figure 4). The results confirmed the RNA-seq data that the four *BnaCRF8* genes were upregulated by P deficiency and had stronger expression levels in the leaves than in the roots under P deficiency. Taken together, these results imply that the four *BnaCRF8* genes might participate in the growth regulation of plants in response to P deficiency. Furthermore, pairwise alignment analyses showed that the amino acid identities of the four *BnaCRF8* genes were more than 80% (Figure S4), implying their conserved roles in the P starvation response.

### 2.4. Subcellular Localization of the BnaCRF8 Genes

**Figure 5:**
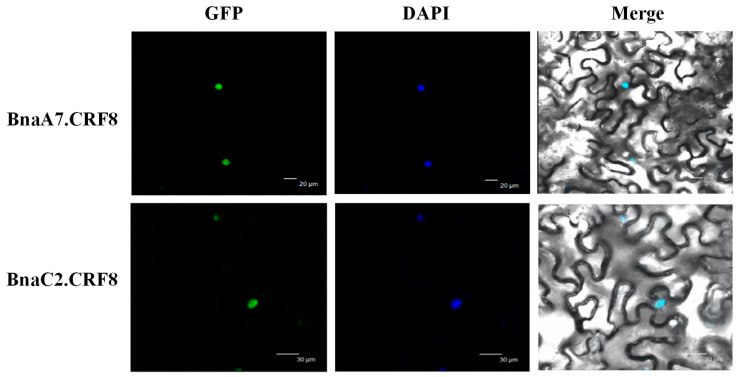
Subcellular localization of BnaA7.CRF8 and BnaC2.CRF8. Tobacco leaves were infiltrated with *Agrobacterium* harboring *35S::BnaA7.CRF8-GFP* and *35S::BnaAC2.CRF8-GFP*, respectively. DAPI (4′,6-diamidino-2-phenylindole) staining was used to indicate the positions of nuclei. GFP, green fluorescent protein.

In *B. napus,* we performed the subcellular localization analyses of BnaCRF proteins using the Wolf Psort program. The result showed that most of the BnaCRF proteins, including the four CRF8s, were predicted to be nuclear localized (Table S1). In line with this prediction, the CRF8 protein identified in *Arabidopsis* is localized in the nucleus as well [19]. To investigate the physiological roles of BnaCRF8 proteins in Pi homeostasis, BnaA7.CRF8 and BnaC2.CRF8 were further randomly selected to be experimentally examined for their subcellular localization in the plant cell. When transiently expressed in tobacco leaves, both BnaA7.CRF8-GFP and BnaC2.CRF8-GFP fusion protein signals were completely merged with the DAPI (4′,6-diamidino-2-phenylindole, a blue-fluorescent DNA stain) signal (Figure 5). This result demonstrates that BnaCRF8 proteins localize in the nucleus.

### 2.5. Phenotypical Characterization of the BnaCRF8 Overexpression Lines in Arabidopsis under Varied Phosphate Conditions

**Figure 6:**
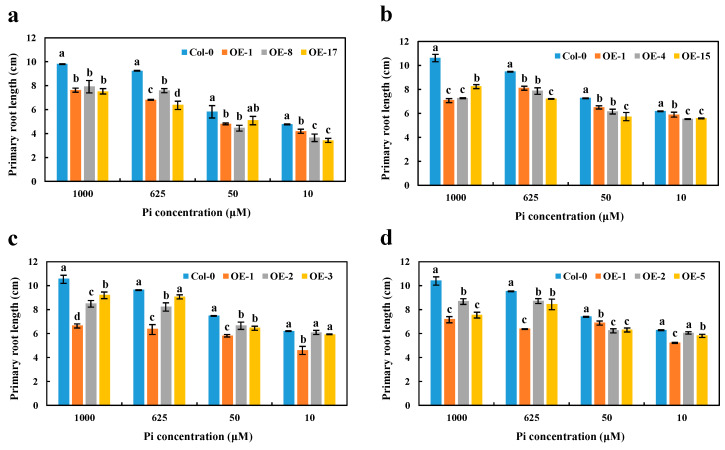
Primary root length comparison of the overexpression (OE) *BnaCRF8* lines and the wild type (Col-0) at varied Pi conditions. Eleven-day-old plants grown on 10, 50, 625, and 1000 μM Pi were imaged for primary root length measurement. (**a**) OE *BnaA7.CRF8* lines; (**b**) OE *BnaC2.CRF8* lines; (**c**) OE *BnaA2.CRF8* lines; (**d**) OE *BnaCn.CRF8* lines. Statistics were performed by Duncan’s test at the significance level of *p* < 0.05. Values represent mean ± SD, n = 4 replicates of 20 seedlings each. Small letters (a, b, c, and d) above the bars indicate mean with significant differences at *p* < 0.05 by Duncan’s test.

Given the nuclear localization and strong Pi-deficiency response of the *BnaCRF8* genes, we further investigated their physiological roles in Pi homeostasis. To established transgenic lines of each *BnaCRF8* in *Arabidopsis*, the 35S promoter was used to control their constant expression levels. Three independent *T3* homozygous lines of each OE *BnaCRF8* were selected to characterize the function in Pi homeostasis (Figure S5). PR and LR are two visible robust parameters of Pi homeostasis. Thus, the phenotypical parameters of 11-day-old seedlings grown in 10, 50, 625, and 1000 μM Pi were analyzed and the results were analyzed statistically. OE of *BnaA7.CRF8* in *Arabidopsis* developed shorter PR compared with the wild type (Col-0) regardless of the Pi concentrations (Figure 6a and Figure S6). The shorter PR was also observed in OE *BnaC2.CRF8* lines at any Pi concentrations (Figure 6b and Figure S7). Consistently, all OE *BnaA2.CRF8* lines and OE *BnaCn.CRF8* lines developed shorter PR compared with Col-0 plants at 50, 625, and 1000 μM Pi, as well as some individual lines at 10 μM Pi (Figure 6c,d and Figure S7). Thus, these results suggest that *BnaCRF8* genes play a negative role in PR growth under varied Pi levels.

**Figure 7:**
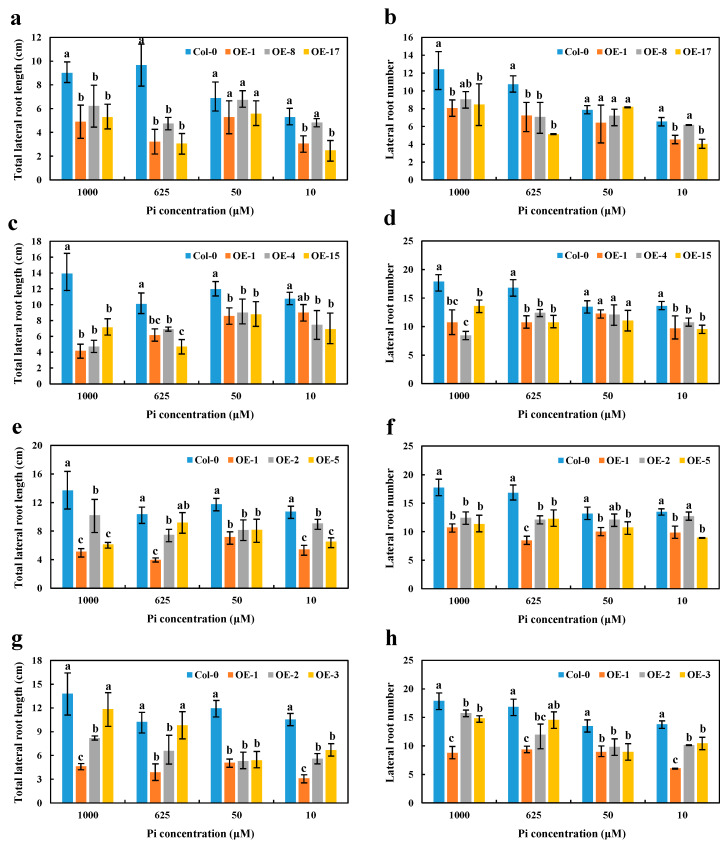
Comparison of the lateral root number and lateral root length among the OE *BnaCRF8* lines and Col-0 at varied Pi conditions. Eleven-day-old plants grown on 10, 50, 625, and 1000 μM Pi were imaged for measurement of the lateral root number and length. (**a**,**b**) OE *BnaA7.CRF8* lines; (**c**,**d**) OE *BnaC2.CRF8* lines; (**e**,**f**) OE *BnaCn.CRF8* lines; (**g**,**h**) OE *BnaA2.CRF8* lines. Statistics were performed by Duncan’s test at the significance level of *p* < 0.05. Values represent mean ± SD, n = 4 replicates of 20 seedlings each. Small letters (a, b, and c) above the bars indicate mean with significant differences at *p* < 0.05 by Duncan’s test.

High Pi concentrations (625 and 1000 μM) significantly disrupted the growth and reduced the number of LR in OE *BnaA7.CRF8* lines compared with Col-0 plants. However, relatively low Pi concentrations (50 and 10 μM) had little effect on the growth and number of LR between OE *BnaA7.CRF8* and Col-0 plants (Figure 7a,b). Interestingly, only the LR number was almost equal among OE *BnaC2.CRF8* lines, OE *BnaCn.CRF8* lines, and Col-0 plants at 50 μM Pi, and these *BnaC2.CRF8* lines and *BnaCn.CRF8* lines had worse growth and a reduced number of LR relative to Col-0 plants at any Pi concentrations (Figure 7c–f). The constant negative regulation of the LR growth and number was observed in the OE *BnaA2.CRF8* lines at all Pi concentrations tested (Figure 7g,h). These results suggest that OE of *BnaCRF8* genes negatively regulate the LR morphogenesis of the plants in response to the Pi dynamic. It is reasonable that the root function weakened by the expression of *BnaCRF8* genes should hinder the shoot growth. With the exception of OE *BnaCn.CRF8* at 10 μM Pi, the fresh weights of the OE *BnaCRF8* lines were totally lower than that of Col-0 at all Pi conditions tested (Figure S8).

### 2.6. BnaA7.CRF8 is a Negative Regulator of Phosphate Homeostasis in Arabidopsis

**Figure 8:**
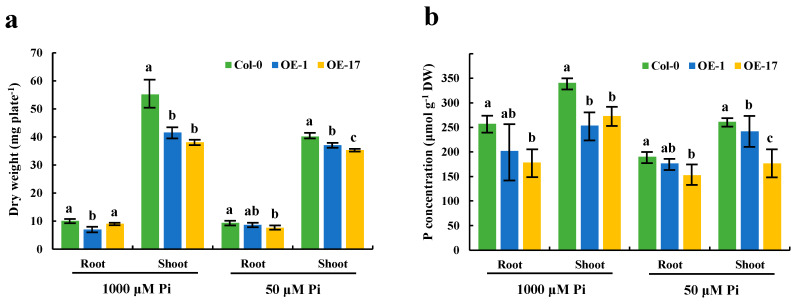
Effects of different phosphate (Pi) concentrations on the dry weight and endogenous Pi levels of the OE *BnaA7.CRF8* lines and Col-0 plants. Eleven-day-old OE *BnaA7.CRF8* lines and Col-0 plants grown on 50 and 1000 μM Pi were imaged for measurement of the dry weight (**a**) and endogenous Pi levels (**b**). Values represent mean ± SD, n = 4 replicates of 20 seedlings each. Small letters (a, b, and c) above the bars indicate mean with significant differences at *p* < 0.05 by Duncan’s test.

**Figure 9:**
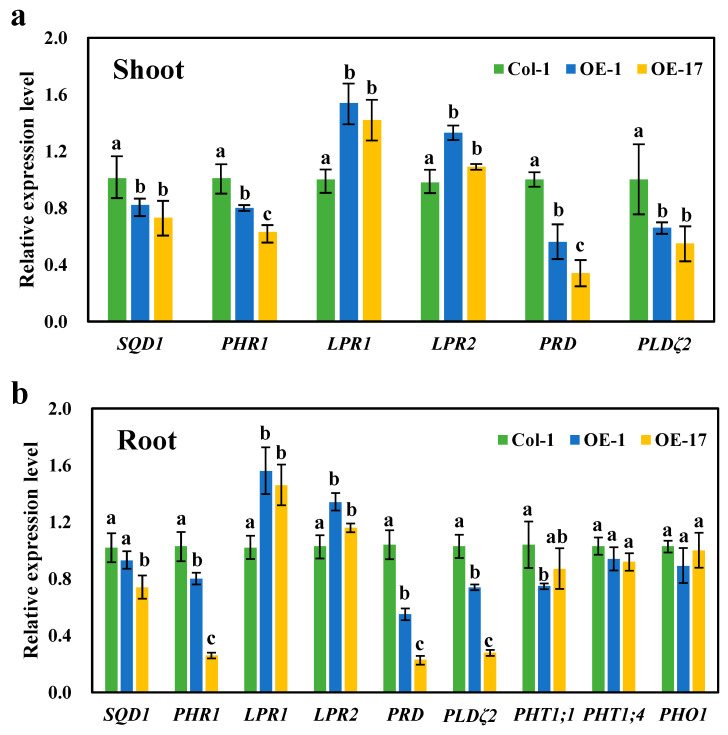
Relative expression levels of the Pi starvation-responsive genes in the wild-type (Col-0) and overexpression *BnaA7.CRF8* lines. Shoots (**a**) and roots (**b**) of the 11-day-old plants grown on 1000 μM Pi were used to perform qRT-PCR analyses. Data represent mean ± SD of 3 biological replicates with 20 plants each. Different letters (a, b, and c) indicate mean with significant differences at *p* < 0.05 by Duncan’s test. *LPR1*/*2*, *LOW PHOSPHATE ROOT1*/*2*; *PHO1*, *PHOSPHATE1*; *PHR1*, *PHOSPHATE STARVATION RESPONSE1*; *PHT*, *PHOSPHATE TRANSPORTER*; *PRD*, *PHOSPHATE ROOT DEVELOPMENT; PLDζ2* encodes an enzyme in phosphatidylcholine hydrolysis; *SQD1* encodes an enzyme in sulfolipid biosynthesis.

It is evident that the expression of the *BnaCRF8* genes limited root development to a larger extent at high Pi concentrations (625 and 1000 μM) than at low Pi concentrations (50 and 10 μM). We speculated that the Pi homeostasis underwent stronger disruption by *BnaCRF8* gene expression at high Pi concentrations compared with low Pi concentrations. To this end, the OE *BnaA7.CRF8* lines and Col-0 were used for further investigation at 1000 and 50 μM Pi, which represent high-Pi and low-Pi treatments, respectively. There was a great reduction in the dry weight (DW) of OE *BnaA7.CRF8* roots compared with Col-0 roots at 50 μM Pi, and it was even larger at 1000 μM Pi (Figure 8a). The Pi concentrations of OE *BnaA7.CRF8* lines were 81.8%–92.7% of Col-0 in roots and were 68.9%–93.3% of Col-0 in shoots at 50 μM Pi (Figure 8b). The Pi concentrations of OE *BnaA7.CRF8* lines were 68.8%–78.5% of Col-0 in roots and were 75.1%–80.9% of Col-0 in shoots at 1000 μM Pi (Figure 8b). However, we could conclude that the changes in the Pi concentrations between the OE *BnA7.CRF8* lines and Col-0 plants at 1000 μM Pi were larger than that at 50 μM Pi statistically. Moreover, the minimum concentration of P among all tested samples was 158 μM g^−1^ DW in the roots of the #17 OE *BnaA7.CRF8* line at 50 μM Pi (Figure 8b), which is a sufficient P demand for plant growth. Thus, the Pi concentration reduction is not likely causal for the growth inhibition of the OE *BnaA7.CRF8* lines. This is further supported by the comparable expression levels of Pi uptake transporters *PHT1;1*, *PHT1;4*, and *PHO1* between Col-0 roots and OE *BnaA7.CRF8* roots (Figure 9). Interestingly, the expression levels of *LPR1*/*LPR2* and *PRD* were significantly higher and lower in OE *BnaA7.CRF8* than in Col-0, respectively (Figure 9). *LPR1*/*LPR2* trigger reactive oxygen species (ROS) to inhibit root tip growth under a low-Pi condition, and the *PRD* gene is involved in the regulation of root architectural responses to Pi starvation by controlling PR and LR elongation (Table 1). In addition, the maintenance of Pi homeostasis requires components, such as membrane lipid remodeling factors *SQD1*, *PLDζ2*, and the major regulator *PHR1* (Table 1), all of which were suppressed in OE *BnaA7.CRF8* roots relative to Col-0 roots (Figure 9). These results suggest that *BnaA7.CRF8* might have a negative effect on Pi homeostasis without the direct regulation of Pi uptake in plants.

## 3. Discussion

TFs are the most central regulator to transduce environmental stresses to activate adaptive gene networks in plants. Pi deficiency is a common abiotic stress in agricultural production, and has gained much research attention by botanists and biologists over the last decades in order to improve plant growth in response to Pi deprivation [2]. Based on the transcriptomic analyses, thousands of differentially expressed genes were identified, including many TFs, such as NAC (NAM, ATAF1/2, CUC2), MYB (v-myb avian myeloblastosis viral oncogene homolog), AP2/ERF, C2H2 (Cys2/His2 zinc finger protein), bHLH (basic helix-loop-helix protein), WRKY (pronounced ‘worky’, WRKY domain containing protein), and CCAAT-binding families [25,26,27,28,29]. The *CRF* family is a small subset of the AP2/ERF TF superfamily and was identified in *Arabidopsis* and other plants [18,30]. In *B. napus*, a total of 40 *CRF* genes were identified previously [22]. In this study, four novel *CRF* genes (Figure 1 and Table S1) were found based on the genome-wide dissection of *B. napus* using *Arabidopsis CRF* family information. Since the conserved CRF-specific motif was not evident in three novel BnaCRF proteins (Figure 2 and Figure S1), they have likely been ignored in previous identification using the CRF domain as a search query [22]. *Brassica* crops have a bigger biomass compared with *Arabidopsis*, and a sophisticated stress response system is always necessary to fine-tune their development and growth. The frequential expansion of genes through gene duplication, such as the greater number of *CRF* genes in *B. napus* relative to *Arabidopsis*, is an efficient adaption strategy in an ever-changing environment.

Not all *CRF* genes are cytokinin responsive, but a range of environmental stimuli, including biotic and abiotic stresses, are the cues [19,30,31,32]. In *Arabidopsis*, *CRF* genes play broader roles in reproductive development, embryogenesis, root architecture, and abiotic stress adaption [19,20,32,33,34,35]. For example, *AtCRF4* is rapidly upregulated after exposure to high-nitrogen (N) conditions, and OE of *AtCRF4* largely disrupts N homeostasis and reduces growth in *Arabidopsis* [36], as *AtCRF4* controls several key N-responsive TFs [24]. *AtCRF8* is specially induced in Pi-deprived shoots and roots, and RNA interference of *AtCRF8* results in augmented LR and Pi accumulation in plants, while OE of *AtCRF8* limits root growth [19]. In line with these results, all the *CRF8* genes are strongly induced by Pi deficiency in *B. napus* leaves and roots (Figure 3), and OE *BnaCRF8* lines tend to reduce PR growth, LR number and elongation, and Pi concentration from low-Pi to high-Pi conditions (Figure 6, Figure 7 and Figure 8). The Pi concentration reduction in the OE *BnaA7.CRF8* lines could not be ascribed to the Pi transporters because their transcription activities were not altered between OE *BnaA7.CRF8* lines and Col-0 plants (Figure 9). Expression of *AtPHR1* is constitutive under different Pi conditions and the *Arabidopsis* mutant *phr1* showed impaired expression of most PSR genes and Pi allocation [14]. It is a possibility that the downregulation of *PHR1* results in low Pi uptake in OE *BnaA7.CRF8* lines (Figure 8 and Figure 9). The limited growth of PR and LR by OE of *BnaA7.CRF8* contributes to the decrease in root surface area, which probably downregulates the Pi uptake efficiency as well. Decreased expression of *PHR1* in OE *BnaA7.CRF8* lines presumably alters the expression profile of a series of PSR genes. Further investigation is required to assess the relationship between *PHR1*-mediated PSR genes and root architecture alternation in OE lines.

Expression of *BnaA7.CRF8* modulated several PSR genes in the roots, leading to negative effects on Pi homeostasis (Figure 9). The *Arabidopsis* mutants *lpr1*, *lpr2*, and *lpr1/lpr2* are insensitive to low Pi stress with a longer PR length than that of the Col-0 plants [12], while OE of *LPR1* reduces the PR length of *Arabidopsis* compared to the control plant [37]. It has been demonstrated that *LPR1*-meidated ROS production is essential for the inhibition of PR growth under Pi deficiency [37]. In OE *BnaA7.CRF8* lines, the mRNA abundances of *LPR1* and *LPR2* were higher than that of Col-0 (Figure 9), suggesting that increased ROS might accumulate in the OE *BnaA7.CRF8* lines. *Arabidopsis* with functional disruption of *PRD* has less-developed LR and PR under Pi deficiency, but the expression levels of *PHT1;1* and *PHT1;4* are similar to that of Col-0 plants [13]. In agreement with this, the expression levels of Pi transporter genes, including *PHT1;1*, *PHT1;4*, and *PHO1*, were similar between the OE *BnaA7.CRF8* lines and Col-0 plants, and the expression of the *PRD* gene was lower in the OE *BnaA7.CRF8* lines relative to Col-0 plants (Figure 9). This result suggests that the decreased expression of the *PRD* gene might inhibit root architecture development in the OE *BnaA7.CRF8* lines. Another well-known adaptive mechanism to P depletion in plants is lipid remodeling. Under Pi-stress conditions, plants can replace membrane phospholipids to P-free sulfolipids and galactolipids to save Pi for vital metabolisms [3]. Genes involved in these processes, such as *SQD1*/*2*, *DGD1*/*2*, and *PLDζ1*/*2*, are found to be upregulated by Pi starvation [3,9,10,11]. However, the expression levels of *SQD1* and *PLDζ2* genes were inhibited by the overexpression of *BnaA7.CRF8* in *Arabidopsis.* The reduced expression of the *SQD1* and *PLDζ2* genes in the OE *BnaA7.CRF8* lines indicates a low sulfolipid accumulation level and slow hydrolysis of phosphatidylcholine, which are obviously detrimental for Pi homeostasis. We noted that *BnaCRF8* genes had a stronger low-Pi response in shoots than that in roots (Figure 4), and the DW of OE *BnaA7.CRF8* lines was reduced significantly relative to Col-0 under 50 and 1000 μM Pi (Figure 8). This implies that *BnaCRF8* genes may play more important roles in Pi homeostasis in shoots than in roots. Except for *BnaC2.CRF8* in the roots of *B. napus*, no more than 2-fold changes were detected for the other three genes within 12 h after low-Pi treatment (Figure 4), demonstrating that the transcriptional response of *BnaCRF8* genes to low Pi is not an early event compared to many other TFs that are rapidly induced by low Pi. Transcriptional activities of the *BnaCRF8* genes were intensified upon prolonged Pi-deficiency treatment and were reversibly downregulated after Pi resupply (Figure 4), suggesting that *BnaCRF8* genes mainly participate in the adaption response to Pi deficiency at a late stage. It has been believed that the long-term Pi deficiency certainly retarded plant growth and development. Therefore, there must be an efficient strategy employed by plants to cope with continuous Pi stress and to regulate plant growth. Based on the findings in this study, we propose that *BnaCRF8* genes are one kind of central TF genes regulating plant growth and development under continuous Pi deficiency.

Taken together, we identified 44 *CRF* genes, including 4 novel genes, on the whole genome of *B. napus*, and found 4 *BnaCRF8* genes that are strongly upregulated by low Pi stress. Phenotypic characterization of OE *BnaCRF8 Arabidopsis* plants combined with the analyses of Pi homeostasis-related genes and Pi concentrations in plants suggest that the upregulation of *BnaCRF8* genes by low-Pi stress might be a smart adaptive strategy to cope with continuous Pi deficiency in the environment.

## 4. Materials and Methods

### 4.1. Identification of CRF TFs in the Brassica napus Genome

*CRF* genes were identified in *B. napus* based on the 12 CRF protein sequences from *Arabidopsis* [16,18] using the BLASTP search program in the CNS-Genoscope database (http://www.genoscope.cns.fr/brassicanapus/) [21], which provides gene annotation, including the chromosome localization and genomic sequence. Redundant sequences were removed manually. The CRF domain and AP2/ERF domain of all identified *BnaCRF* genes were future analyzed using the SMART database (http://smart.embl-heidelberg.de/) [38], and NCBI Conserved Domain Search database (http://www.ncbi.nlm.nih.gov/Structure/cdd/wrpsb.cgi) [39]. The physicochemical parameters of BnaCRF proteins, including MW and PI, were calculated using the ExPASy tool (http://www.expasy.org/tools/) [40]. Subcellular localization prediction was conducted using Wolf Psort (http://www.genscript.com/wolf-psort.html) [41].

### 4.2. Phylogenetic and Conserved Motif Analyses of BnaCRF Genes

Multiple alignments of candidate CRF protein sequences were carried out using ClustalW with default parameters [42]. An unrooted phylogenetic tree of all CRF proteins from *B. napus*, *B. rapa*, *B. oleracea*, and *Arabidopsis* were generated with MEGA6 [43] using the NJ method with the following parameters: Poisson correction, complete deletion, and 1000 bootstrap replicates. Conserved motifs in *B. napus* were identified using the motif-finding tool MEME (Multiple EM for Motif Elicitation, V 4.11.2) [23]. MEME searching was performed across BnaCRF protein sequences using the following parameters: (1) Optimum motif width was set to ≥6 and ≤100; (2) the maximum number of motifs was set to identify 10 motifs; and (3) the others were the default settings.

### 4.3. Chromosomal Location and Gene Duplication Analyses

Physical location information of the *BnaCRF* genes was retrieved from the CNS-Genoscope genome database, and was mapped to rapeseed chromosomes using Circos [44]. Gene duplication events and collinearity relationships were analyzed using the Multiple Collinearity Scan toolkit (MCScanX) [45]. The criteria for analyzing potential gene duplications were: (a) Length of alignable sequence covers >75% of the longer gene, and (b) similarity of aligned regions >75%.

### 4.4. Plant Materials and Treatments

The P-efficient *B. napus* genotype ‘Eyou Changjia’ [46] and *Arabidopsis* Col-0 ecotype were used in this study. Plants were cultured in a hydroponic condition in an illuminated culture room with a cycle of 16 h/24 °C day and 8 h/22 °C night, and a light intensity of 300–320 μmol proton m^−2^ s^−1^. The modified Hoagland’s solution was used for plant growth [47]. The surface-sterilized seeds were immersed for 24 h in deionized water prior to germination on moistened gauze. Six-day-old seedlings were transferred into a 10-L black plastic container filled with quarter-strength nutrient solution for 5 day’s growth followed by half strength and full strength with an interval of 5 days. Seedlings were fixed on a perforated foam board with sponge. For the phenotypic characterization, *Arabidopsis* were grown vertically on solid medium (1.5% (*w*/*v*) sucrose and 0.9% (*w*/*v*) agar with 10, 50, 625, and 1000 μM Pi in Petri dishes).

For RNA-seq analyses in *B. napus*, 15-day-old seedlings were transferred to nutrient solutions with 0 mM or 250 μM Pi for 10 days. The leaves and roots were harvested for RNA extraction, respectively. For the qRT-PCR assay in *B. napus*, 15-day-old seedlings were treated with P-free nutrient solutions. Samples were harvested at 0, 12, 72, and 240 h after P-free treatment, respectively. After 10 days’ treatment, Pi recovery (250 μM KH_2_PO_4_) was added to the nutrient solution for 6 and 72 h. Three biological replicates with 4 plants each were included. For the qRT-PCR assay in *Arabidopsis*, the shoots and roots of the 11-day-old plants grown on 1000 μM Pi were harvested for RNA extraction. Three biological replicates with 20 plants each were included. All samples were stored at −80 °C prior to RNA extraction.

### 4.5. RNA Isolation and Real-Time Quantitative PCR

Total RNA was isolated using the total RNA kit (Invitrogen, Carlsbad, CA, USA) according to the manufacturer’s instructions. First-strand cDNA was synthesized using the Prime Script RT reagent Kit (Promega, Madison, WI, USA). Real-time RT-PCR was performed using ABI7300 Real-time Detection System (Applied Biosystems, Foster City, CA, USA) with SYBR Premix Ex TaqTM II (Toyobo, Shanghai, China). The PCR conditions were set as follows: 95 °C for 2 min; 40 cycles of 95 °C for 30 s and 60 °C for 30 s; and 72 °C for 1 min. The 2*^−∆∆CT^* method was used to calculate the relative expression levels of *BnaCRF8* genes using *Actin* as the reference gene. The primers of the 4 *BnaCRF8* genes and the *Actin* gene used for qRT-PCR detection are listed in Table S2.

### 4.6. Expression Pattern Analysis of BnaCRF Genes in Response to Pi Starvation using RNA-Seq Data

The fully expanded leaves and roots of seedlings treated with 0 mM or 250 μM Pi were harvested separately for RNA extraction using TRIzol reagent according to the manufacturer’s instructions (Invitrogen, Carlsbad, CA, USA). Three biological replicates with 4 plants each were included. The quality and integrity of the total RNA were assessed using a NanoDrop 2000 spectrophotometer (Thermo Fisher Scientific, Waltham, MA, USA). Then, 12 RNA samples were subjected to an Illumina Hiseq 2000 platform (Illumina, San Diego, CA, USA) with 100-bp paired-end reads. Finally, a total of 643,846,484 raw reads and 571,929,682 clean reads were generated with an average of ~5.0 Gb sequencing data for each sample. Sequencing reads were aligned to the *B. napus* reference genome [21] with bowtie v2.0.6 and then assembled using TopHat v2.0.12 [48]. The mapped reads were 496,073,698 and the unique mapped reads were 430,982,901 (75.40%). The transcript abundance of each gene was estimated using FPKM based on the length of the gene and reads count mapped to this gene. Differently expressed genes between +P (250 μM Pi) and −P (0 mM Pi) treatments were filtered using the Benjamini and Hochberg’s approach by controlling the false discovery rate (FDR) of 0.05.

### 4.7. Vector Construction and Arabidopsis Transformation

All PCR amplifications of the 4 *BnaCRF8* genes were conducted using Phusion Polymerase (New England Biolabs (Beijing) Ltd., Beijing, China) with the specific primers (Table S2). The PCR reaction product was digested and ligated into EcoRI/XhoI sites of the binary vector pBin35SRed to generate a *35S::BnaCRF8* construct. *Arabidopsis* Col-0 grown on vermiculite to the bud stage was used, and genetically transformed *Arabidopsis* were produced using the *Agrobacterium tumefaciens*-mediated method [49]. The *T3* homozygous generation was established due to the DsRed fluorescence observation [50].

### 4.8. Transient Expression Assays in Nicotiana benthamiana

To construct *35S::BnaA7.CRF8*-GFP and *35S::BnaA2.CRF8*-GFP, the open reading frame sequences of *BnaA7.CRF8* and *BnaA2.CRF8* were fused in frame into the position between the 35S promoter and green fluorescent protein (GFP) sequence in pMDC83 plasmids. An *Agrobacterium* carrying GV3101 construct was harvested by centrifugation and suspended in the solutions containing 10 mM MES, pH 5.6, 10 mM MgCl_2_, and 200 mM acetosyringone to an optical density (600 nm) of 0.8, incubated at room temperature for 3 h, and then used to infiltrate the leaves of tobacco (*Nicotiana benthamiana*) using a needle-free syringe. *Nicotiana benthamiana* was grown in a chamber with a 14 h/25 °C day and 10 h/20 °C night cycle, and a light intensity of ~200 μmol proton m^−2^ s^−1^. After 2 days’ growth, the cellular localization of BnaCRF8-GFP protein in the infiltrated leaves was checked using a Leica SP8 lazer confocal microscopy system (Leica Microsystem, Wetzlar, Germany). DAPI was used to stain the nucleus. The wavelength sets were GFP (excitation: 488 nm, emission: 505–545 nm), DAPI (excitation: 405 nm, emission: 415–450 nm). The images were recorded by the Leica SP8 system and data were further analyzed using ImageJ software (http://rsb.info.nih.gov/ij/).

### 4.9. Measurements of Root Traits

After harvesting, the roots of *Arabidopsis* were scanned at 600 dpi with a scanner. The different root traits, including PR length, LR number, LR length, and total root length, were evaluated using the root image analyses software WinRHIZO Pro (Regent Instruments, Montreal, QC, Canada). Four replicates with 20 plants each were analyzed.

### 4.10. Quantification of Total Phosphorus

Leaves and roots of 11-day-old *Arabidopsis* plants treated with different Pi concentrations were sampled, and then dried at 65 °C to a constant weight. Then, the samples were pre-digested in glass tubes with H_2_SO_4_ for 12 h followed by H_2_O_2_ addition at 180 °C until the solution became colorless. The digestion was continued for another 30 min. The Pi concentration in the samples was quantified as described previously [7]. Four replicates with 20 plants each were analyzed.

### 4.11. Statistical Analyses

Statistics were performed by Duncan’s test at the significance level of *p* < 0.05.

## Figures and Tables

**Table 1:** The molecular functions of the genes analyzed in this study.

Gene Name	Molecular Function	References
*PHT1;1*	Pi uptake	[5]
*PHT1;4*	Pi uptake	[5]
*PHO1*	Pi loading into xylem	[8]
*SQD1*	Sulfolipid biosynthesis under Pi limitation	[9]
*PLDζ2*	Phosphatidylcholine hydrolysis and DGDG accumulation under Pi stress	[11]
*LPR1/LPR2*	Root growth regulation under Pi stress	[12]
*PRD*	Root growth regulation under Pi stress	[13]
*PHR1*	A central transcription factor involved in Pi starvation signaling	[14]

DGDG, digalactosyldiacylglycerol.

## Supplementary Materials

Supplementary materials can be found at https://www.mdpi.com/1422-0067/21/10/3660/s1, Figure S1: Conserved domain analyses of *CRF* genes in *Brassica napus*. **a**, Sequence alignment of conserved CRF domain in *B. napus* and *Arabidopsis thaliana*; **b**, Sequence alignment of conserved the AP2/ERF domain in *B. napus* and *A. thaliana*; **c**, The sequence information of the CRF domain and AP2/ERF domain; **d**, The sequence information of 10 motifs in Figure 2, Figure S2: Chromosomal localization of 44 *CRF* genes in *Brassica napus* genome. Chromosome name is written as A01–A10 and C01–C09. The size of each chromosome was estimated as Mb on the bottom of each chromosome. Bar = 10 Mb, Figure S3: Gene duplication of *BnaCRF* genes in *Brassica napus* genome. The genome visualization tool CIRCOS was used to illustrate the chromosomal distribution of *BnaCRF* genes. The chromosome number is shown inside the circle. The chromosomal location of each *BnaCRF* is shown outside of the corresponding chromosome. Duplicated gene pairs are linked by green lines, Figure S4: Pairwise identity matrix of CRF8 subfamily proteins. CRF8 proteins in *Arabidopsis thaliana* (At), *Brassica napus* (Bna), *B. rapa* (Bra) and *B. oleracea* (Bol) were used for pairwise alignment. The amino acid identities (%) are indicated by the number and column, Figure S5: Semi-qRT-PCR analyses of the expression levels of 4 *CRF8* genes in Col-0 and transgenic lines. *Actin* was used as the internal control. All the experiments were repeated twice with identical results, Figure S6: Phenotypical performances of the 11-day-old wild-type (Col-0) and *BnaA7.CRF8* gene overexpression (OE) lines under 1000 μM, 625 μM, 50 μM and 10 μM Pi. OE lines and Col-0 plants were separated by red lines, Figure S7: Phenotypical performances of the 11-day-old wild-type (Col-0) and overexpression (OE) lines (*BnaC2.CRF8*, *BnaCn.CRF8* and *BnaA2.CRF8*) under 1000 μM, 625 μM, 50 μM and 10 μM Pi. OE lines and Col-0 plants were separated by red lines, Figure S8: Fresh weights of the 11-day-old wild-type (Col-0) and 4 *BnaCRF8* genes overexpression lines under different Pi concentrations. Four treatments were performed as 1000 μM, 625 μM, 50 μM and 10 μM Pi. Statistics were performed by Duncan’s test at the significance level of *P* < 0.05. Values represent mean ± SD, n = 4 replicates of 20 seedlings each, Table S1: *CRF* family genes identified in *Brassica napus*, Table S2: Primer sequences used in this study

## Abbreviations

P	Phosphorus
Pi	Phosphate
TF	Transcription factor
OE	Overexpression
PR	Primary root
LR	Lateral root
CRF	Cytokinin Response Factor
AP2	APETALA2
ERF	ETHYLENE RESPONSIVE FACTOR
FPKM	fragments per kilobase of transcript per million fragments mapped
