# Supplementary material for: Genome-Wide Dissection of the CRF Gene Family in Brassica napus Indicates that BnaCRF8s Specifically Regulate Root Architecture and Phosphate Homeostasis against Phosphate Fluctuation in Plants
Source: Int J Mol Sci. 2020 May 22;21(10):3660. doi: 10.3390/ijms21103660 (PMC7279159; doi:10.3390/ijms21103660)
Supplement: Supplementary file 1 [file ijms-21-03660-s001.zip › Supplementary Files/Figures S1-S8.pdf]

Figure S1

a CRF domain

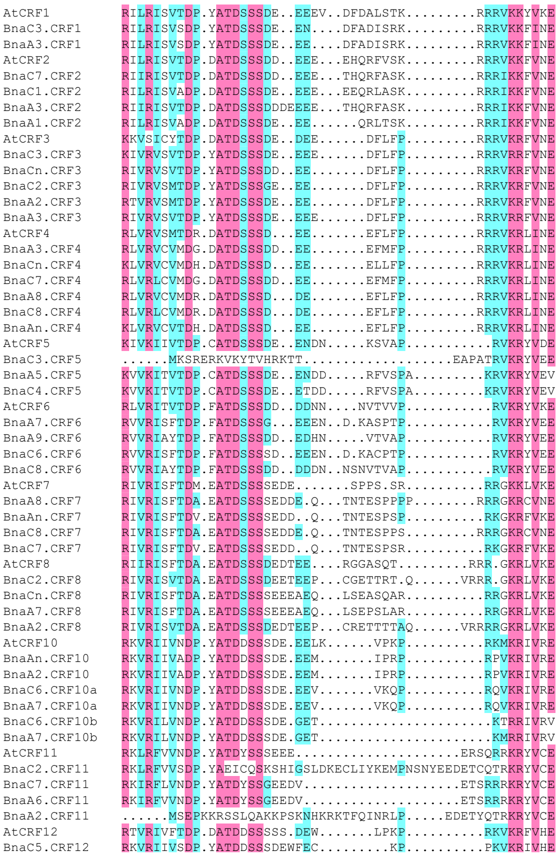

b AP2/ERF domain

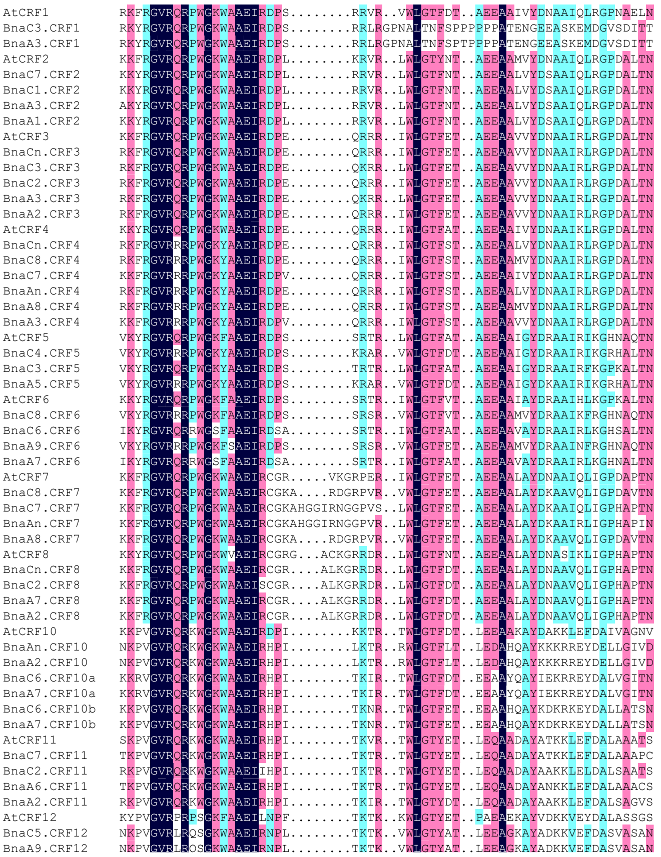

c

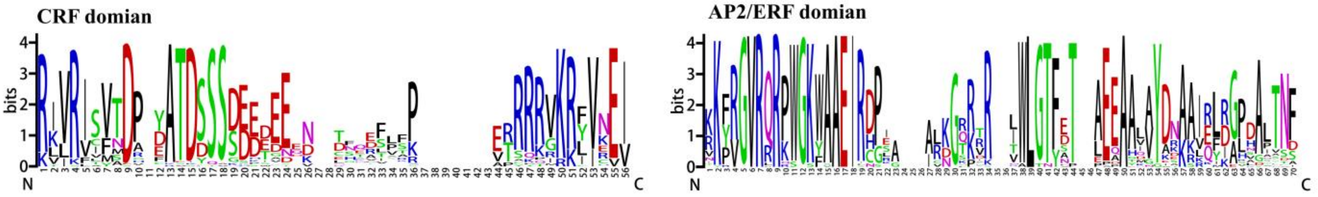

d

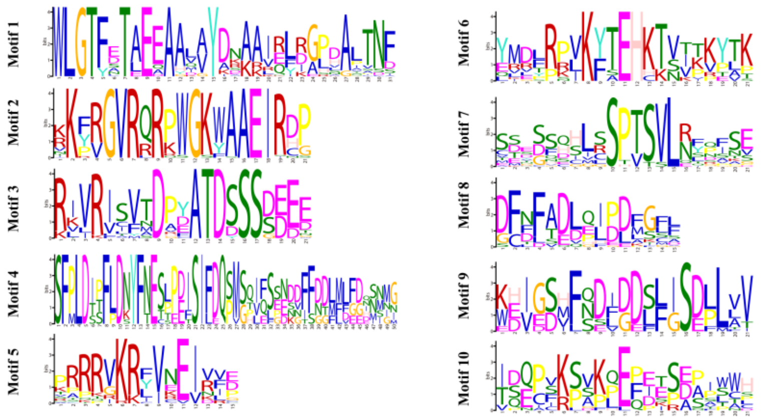

Figure S2

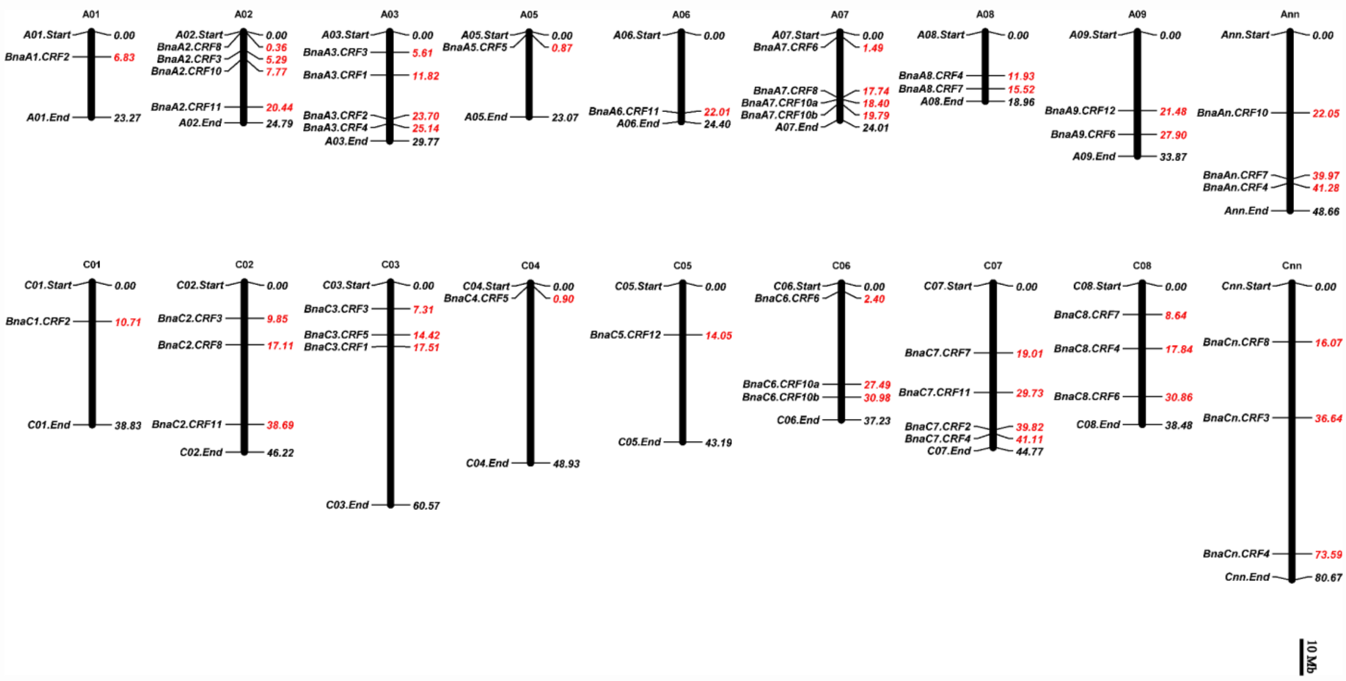
A01
A01.Start 0.00
BnaA1.CRF2 6.83
A01.End 23.27
A02
A02.Start 0.00
BnaA2.CRF8 0.36
BnaA2.CRF3 5.29
BnaA2.CRF10 7.77
BnaA2.CRF11 20.44
A02.End 24.79
A03
A03.Start 0.00
BnaA3.CRF3 5.61
BnaA3.CRF1 11.82
BnaA3.CRF2 23.70
BnaA3.CRF4 25.14
A03.End 29.77
A05
A05.Start 0.00
BnaA5.CRF5 0.87
A05.End 23.07
A06
A06.Start 0.00
BnaA6.CRF11 22.01
A06.End 24.40
A07
A07.Start 0.00
BnaA7.CRF6 1.49
BnaA7.CRF8 17.74
BnaA7.CRF10a 18.40
BnaA7.CRF10b 19.79
A07.End 24.01
A08
A08.Start 0.00
BnaA8.CRF4 11.93
BnaA8.CRF7 15.52
A08.End 18.96
A09
A09.Start 0.00
BnaA9.CRF12 21.48
BnaA9.CRF6 27.90
A09.End 33.87
Ann
Ann.Start 0.00
BnaAn.CRF10 22.05
BnaAn.CRF7 39.97
BnaAn.CRF4 41.28
Ann.End 48.66
C01
C01.Start 0.00
BnaC1.CRF2 10.71
C01.End 38.83
C02
C02.Start 0.00
BnaC2.CRF3 9.85
BnaC2.CRF8 17.11
BnaC2.CRF11 38.69
C02.End 46.22
C03
C03.Start 0.00
BnaC3.CRF3 7.31
BnaC3.CRF5 14.42
BnaC3.CRF1 17.51
C03.End 60.57
C04
C04.Start 0.00
BnaC4.CRF5 0.90
C04.End 48.93
C05
C05.Start 0.00
BnaC5.CRF12 14.05
C05.End 43.19
C06
C06.Start 0.00
BnaC6.CRF6 2.40
BnaC6.CRF10a 27.49
BnaC6.CRF10b 30.98
C06.End 37.23
C07
C07.Start 0.00
BnaC7.CRF7 19.01
BnaC7.CRF11 29.73
BnaC7.CRF2 39.82
BnaC7.CRF4 41.11
C07.End 44.77
C08
C08.Start 0.00
BnaC8.CRF7 8.64
BnaC8.CRF4 17.84
BnaC8.CRF6 30.86
C08.End 38.48
Cnn
Cnn.Start 0.00
BnaCn.CRF8 16.07
BnaCn.CRF3 36.64
BnaCn.CRF4 73.59
Cnn.End 80.67
10 Mb

Figure S3

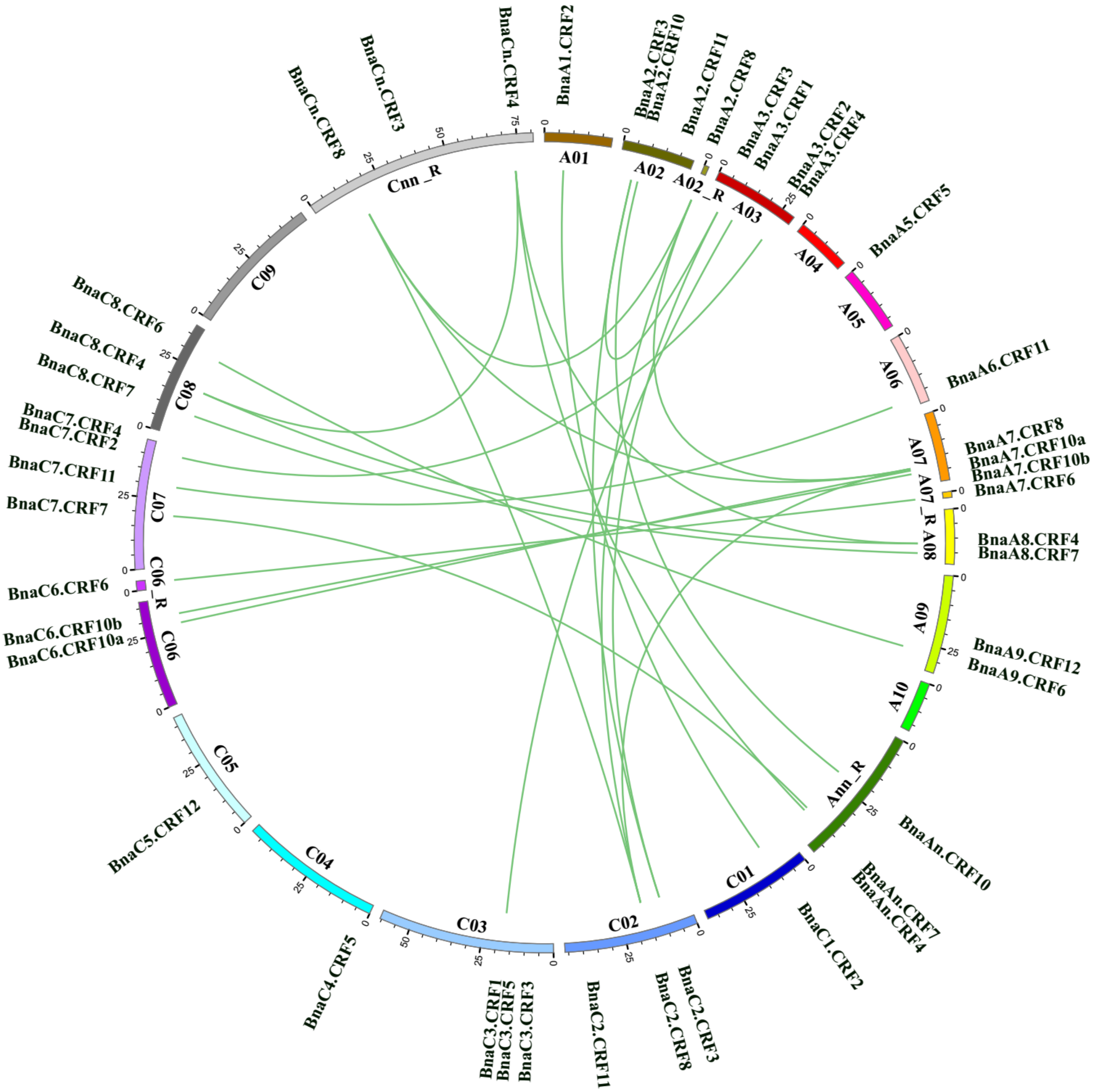

BnaA1.CRF2
BnaA2.CRF3
BnaA2.CRF10
BnaA2.CRF11
BnaA2.CRF8
BnaA3.CRF3
BnaA3.CRF1
BnaA3.CRF2
BnaA3.CRF4
BnaA5.CRF5
BnaA6.CRF11
BnaA7.CRF8
BnaA7.CRF10a
BnaA7.CRF10b
BnaA7.CRF6
BnaA8.CRF4
BnaA8.CRF7
BnaA9.CRF12
BnaA9.CRF6
BnaAn.CRF10
BnaAn.CRF7
BnaAn.CRF4
BnaC1.CRF2
BnaC2.CRF3
BnaC2.CRF8
BnaC2.CRF11
BnaC3.CRF1
BnaC3.CRF5
BnaC3.CRF3
BnaC4.CRF5
BnaC5.CRF12
BnaC6.CRF10b
BnaC6.CRF10a
BnaC6.CRF6
BnaC7.CRF7
BnaC7.CRF11
BnaC7.CRF4
BnaC7.CRF2
BnaC8.CRF7
BnaC8.CRF4
BnaC8.CRF6
BnaCn.CRF8
BnaCn.CRF3
BnaCn.CRF4
A01
A02
A02_R
A03
A04
A05
A06
A07
A07_R
A08
A09
A10
Ann_R
C01
C02
C03
C04
C05
C06
C06_R
C07
C08
C09
Cnn_R

Figure S4

	AtCRF8							
BnaA2.CRF8	75.60	BnaA2.CRF8						
BnaA7.CRF8	77.11	81.66	BnaA7.CRF8					
BnaC2.CRF8	77.58	92.22	82.04	BnaC2.CRF8				
BnaCn.CRF8	77.71	82.84	97.58	82.04	BnaCn.CRF8			
BraA2.CRF8	75.60	100.00	82.25	92.22	82.84	BraA2.CRF8		
BraA7.CRF8	76.51	79.88	98.18	80.84	95.76	79.88	BraA7.CRF8	
BolC2.CRF8	78.66	94.01	83.23	96.95	83.83	94.01	82.04	BolC2.CRF8
BolCn.CRF8	77.71	82.84	97.58	82.04	100.00	82.84	95.76	83.23

Figure S5

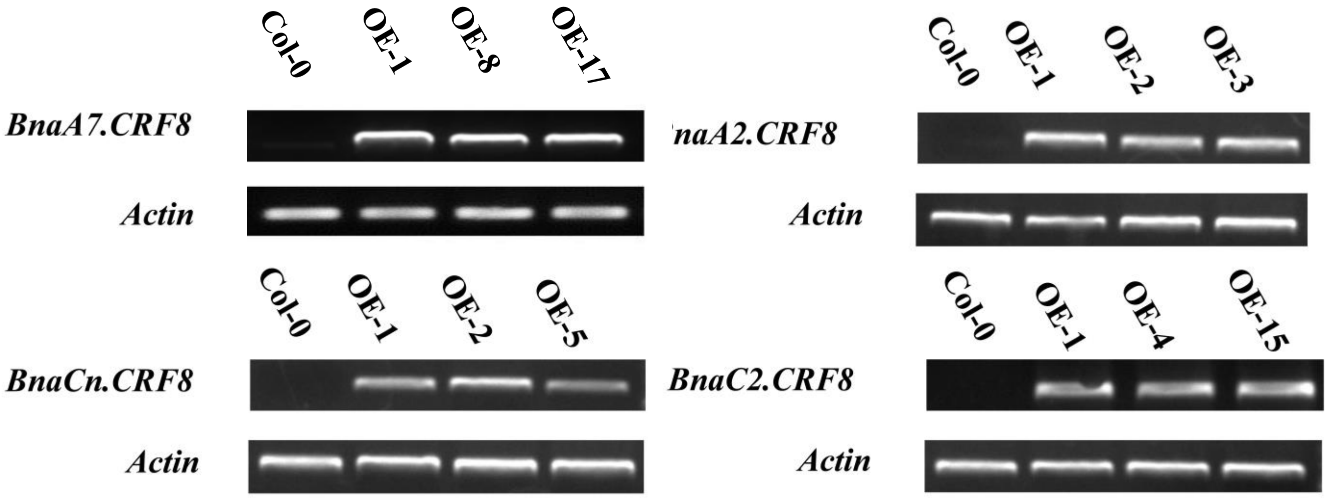

Col-0
OE-1
OE-8
OE-17
BnaA7.CRF8
Actin
Col-0
OE-1
OE-2
OE-3
naA2.CRF8
Actin
Col-0
OE-1
OE-2
OE-5
BnaCn.CRF8
Actin
Col-0
OE-1
OE-4
OE-15
BnaC2.CRF8
Actin

Figure S6

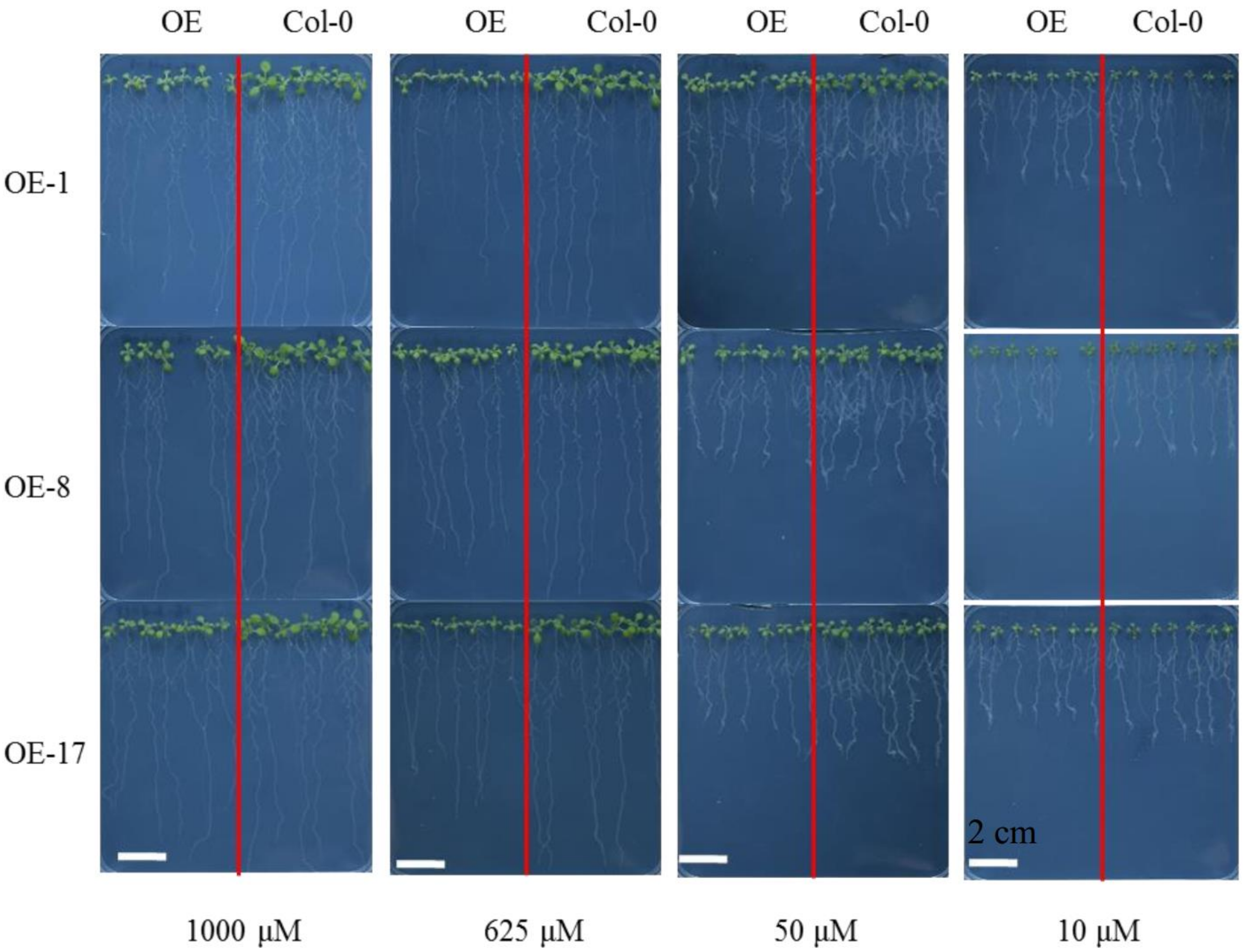
OE
Col-0
OE
Col-0
OE
Col-0
OE
Col-0
OE-1
OE-8
OE-17
2 cm
1000 µM
625 µM
50 µM
10 µM

Figure S7

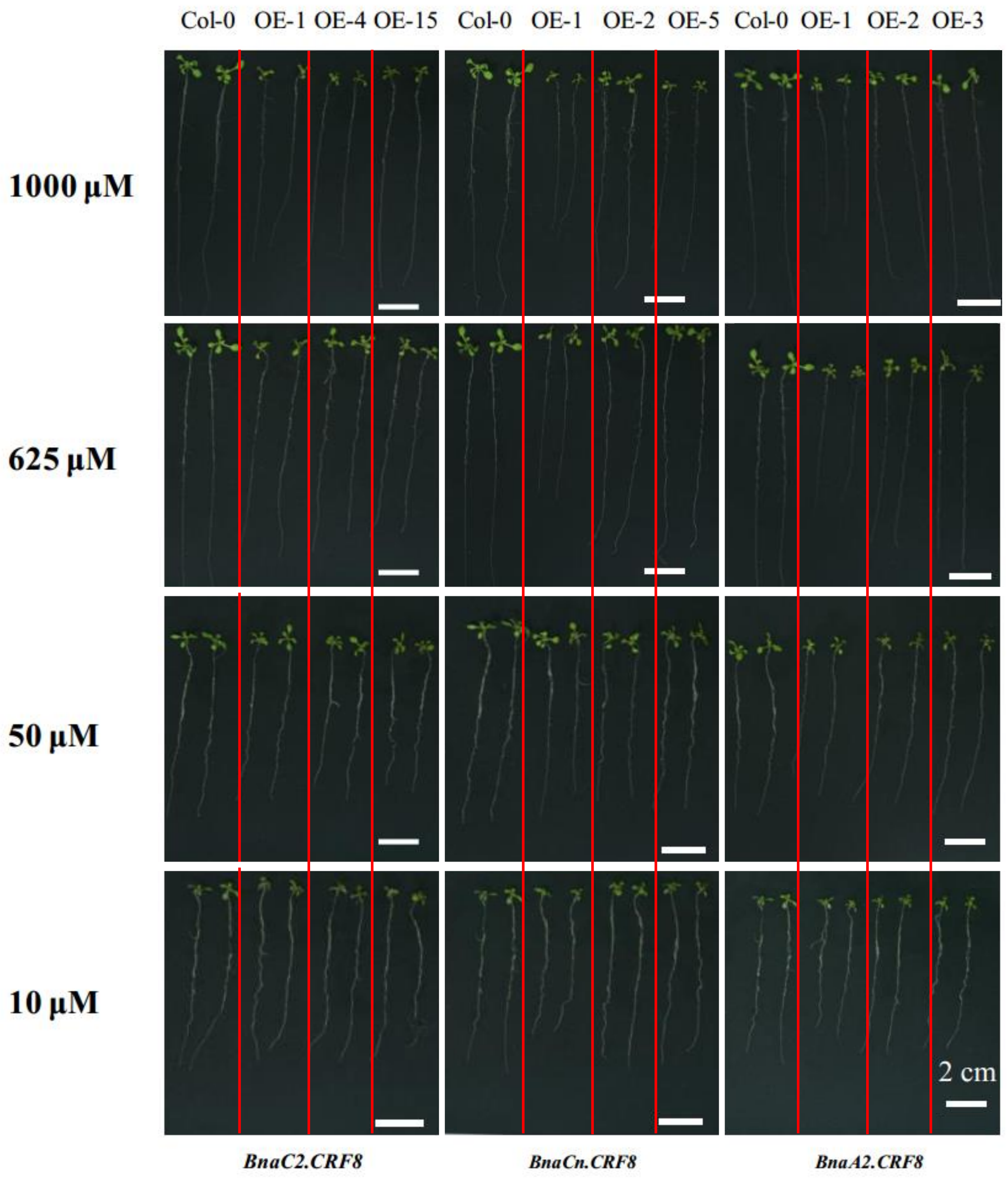
Col-0 OE-1 OE-4 OE-15 Col-0 OE-1 OE-2 OE-5 Col-0 OE-1 OE-2 OE-3
1000 µM
625 µM
50 µM
10 µM
2 cm
BnaC2.CRF8
BnaCn.CRF8
BnaA2.CRF8

Figure S8

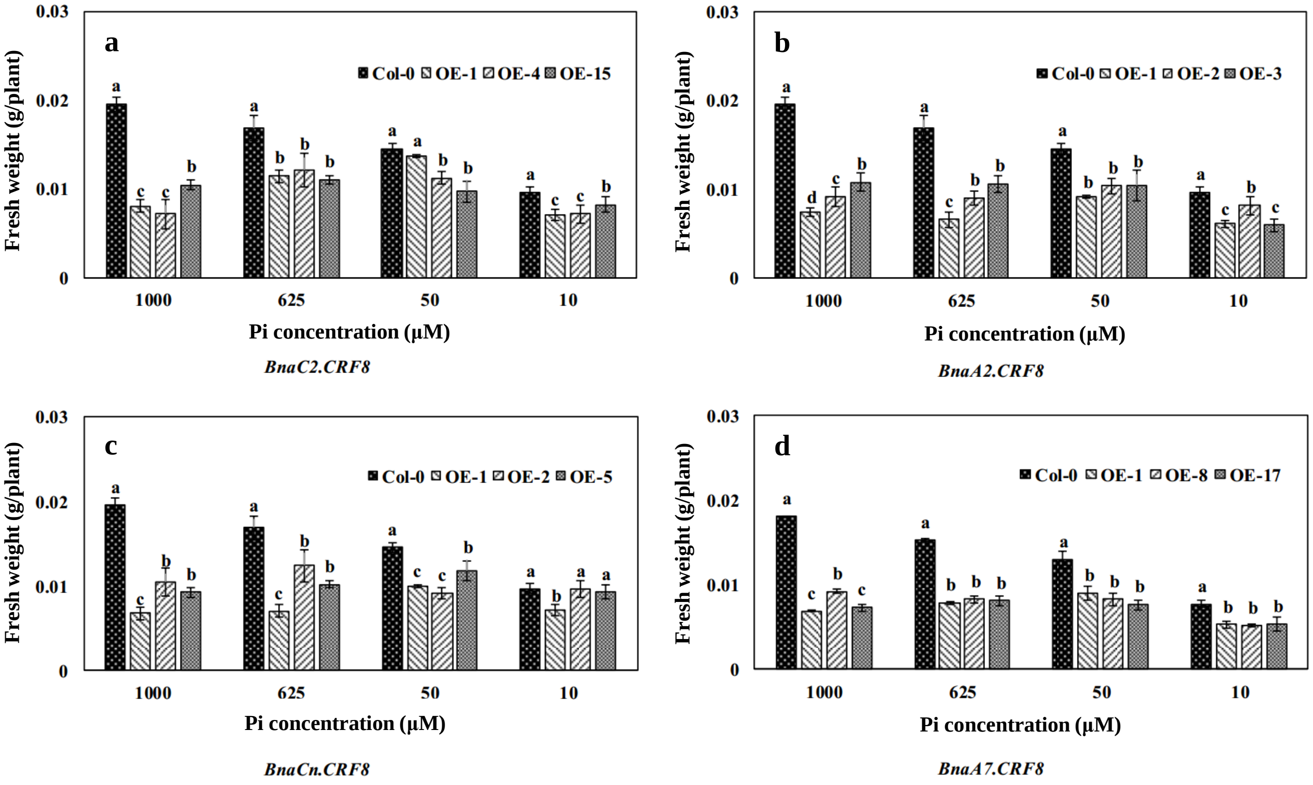
a
Col-0 OE-1 OE-4 OE-15
Fresh weight (g/plant)
Pi concentration (μM)
BnaC2.CRF8
b
Col-0 OE-1 OE-2 OE-3
Fresh weight (g/plant)
Pi concentration (μM)
BnaA2.CRF8
c
Col-0 OE-1 OE-2 OE-5
Fresh weight (g/plant)
Pi concentration (μM)
BnaCn.CRF8
d
Col-0 OE-1 OE-8 OE-17
Fresh weight (g/plant)
Pi concentration (μM)
BnaA7.CRF8
