# Supplementary material for: Genome-Wide Dissection of the CRF Gene Family in Brassica napus Indicates that BnaCRF8s Specifically Regulate Root Architecture and Phosphate Homeostasis against Phosphate Fluctuation in Plants
Source: Int J Mol Sci. 2020 May 22;21(10):3660. doi: 10.3390/ijms21103660 (PMC7279159; doi:10.3390/ijms21103660)
Supplement: Supplementary file 1 [file ijms-21-03660-s001.zip › Supplementary Files/Table S2 Primers.docx]

**Table S2:** Primer sequences used in this study

| Primer name | Forward sequence (5' -3') | Reverse sequence (5' -3') |
| --- | --- | --- |
| Primers used in vector construction | | |
| *OEBnaA2. CRF8* | GAATTCCTCTCTCCCTCCTTTATTACCCC | TCTAGACCATTACAACAGACCTTCATTGC |
| *OEBnaC2. CRF8* | AAGAATTCATGAAGCGTATCGTCAGAATCTCAG | TCTAGACACGACCAACGAAATAACAAAC |
| *OEBnaA7. CRF8* | GCATCTAGAGCATCAAAAGCACTCTCCC | GCTACTCGAGACTACGACAGAGAGAGACCC |
| *OEBnaCn. CRF8* | CGGAATTCGTCTCAGATGAAGCGTATAGTCAG | ATTCTAGACACGAAACCAACGCGCTTC |
| BnaA7. CRF8-GFP | CCTTAATTAAGCCACCATGAAGCGCATCGTCAGAATCTC | AAGGCGCGCCAAGCAACTCGAGCAACGGC |
| Primers used in RT-qPCR analysis | | |
| *BnaA2.CRF8* | CGATCGTCTCTGGCTTGGG | CCACCATCTTCACCTCTTGG |
| *BnaC2. CRF8* | AAATGGGCGGCTGAGATCAGT | GCCACCATCTTGACCTCTTGACA |
| *BnaA7. CRF8* | AATTTCATTCACCGACGCC | TTTGTTGTCGGAATCGGAAG |
| *BnaCn. CRF8* | CTCTTCCGATTCCGACAACAG | AGTTCCCAGCCAAAGACGAT |
| *BnACTIN* | ACAGTGTCTGGATCGGTGGTTC | TGCCTCATCATACTCAGCCTTG |
| *AtACTIN7* | GGAGCTGAGAGATTCCGTTG | GGTGCAACCACCTTGATCTT |
| *AtPHT1;1* | CCTTTGGGTTCCTATATGCG | TAACCTCAGCCTCACCAGAG |
| *AtPHT1;4* | TCAATGGCGTTGCCTTCTGT | ATCACCAAGCCACCCGAAA |
| *AtPHO1* | TGGTTCTCCGGAACAAGAAC | TGACTTCAAGTGACGCCAAG |
| *AtSQD1* | GGGACTCTCAACGTTCTCTTTG | CCCATCGTCCCAAGTTTTAC |
| *AtLPR1* | AGAAACTCCAAAATCAGGGACTACA | ATGGTCCAATATGTGACAATGGTAG |
| *AtLPR2* | GAGGCATAAAGCAAGAGGAGC | GCAGGCAAGTCTCTGTGGAA |
| *AtPRD* | CAAATGCGCTTCGTATCTCC | TAAAACAGCGTCTGCGTCTG |
| *AtPHR1* | GCTTGTCAAGGAATCTGAGGC | GAACTTCCACTCCTGATCTCC |
| *AtPLDζ2* | TTTGAGGACGGTCCAATTGCCA | ACAACACCGATCTCAGAGTCTCGT |
